# Sex-dependent adaptive changes in serotonin-1A autoreceptor function and anxiety in Deaf1-deficient mice

**DOI:** 10.1186/s13041-016-0254-y

**Published:** 2016-08-03

**Authors:** Christine Luckhart, Tristan J. Philippe, Brice Le François, Faranak Vahid-Ansari, Sean D. Geddes, Jean-Claude Béïque, Diane C. Lagace, Mireille Daigle, Paul R. Albert

**Affiliations:** 1 Ottawa Hospital Research Institute (Neuroscience), Ottawa, Canada; 2 Department of Cellular and Molecular Medicine, University of Ottawa Brain and Mind Research Institute, 451 Smyth Road, Ottawa, ON K1H-8M5 Canada

**Keywords:** Major depression, Serotonin, Receptor signaling, Raphe, Anxiety, Transgenerational

## Abstract

The C (-1019) G rs6295 promoter polymorphism of the serotonin-1A (5-HT1A) receptor gene is associated with major depression in several but not all studies, suggesting that compensatory mechanisms mediate resilience. The rs6295 risk allele prevents binding of the repressor Deaf1 increasing 5-HT1A receptor gene transcription, and the Deaf1-/- mouse model shows an increase in 5-HT1A autoreceptor expression. In this study, Deaf1-/- mice bred on a mixed C57BL6-BALB/c background were compared to wild-type littermates for 5-HT1A autoreceptor function and behavior in males and females. Despite a sustained increase in 5-HT1A autoreceptor binding levels, the amplitude of the 5-HT1A autoreceptor-mediated current in 5-HT neurons was unaltered in Deaf1-/- mice, suggesting compensatory changes in receptor function. Consistent with increased 5-HT1A autoreceptor function *in vivo*, hypothermia induced by the 5-HT1A agonist DPAT was augmented in early generation male but not female Deaf1-/- mice, but was reduced with succeeding generations. Loss of Deaf1 resulted in a mild anxiety phenotype that was sex-and test-dependent, with no change in depression-like behavior. Male Deaf1 knockout mice displayed anxiety-like behavior in the open field and light-dark tests, while female Deaf1-/- mice showed increased anxiety only in the elevated plus maze. These data show that altered 5-HT1A autoreceptor regulation in male Deaf1-/- mice can be compensated for by generational adaptation of receptor response that may help to normalize behavior. The sex dependence of Deaf1 function in mice is consistent with a greater role for 5-HT1A autoreceptors in sensitivity to depression in men.

**Electronic supplementary material:**

The online version of this article (doi:10.1186/s13041-016-0254-y) contains supplementary material, which is available to authorized users.

## Introduction

The 5-HT1A receptor is the most abundant and widely expressed serotonin (5-HT) receptor in the brain [1, 2], and functions both as a presynaptic autoreceptor to inhibit firing of raphe 5-HT neurons, and as a post-synaptic receptor that mediates 5-HT actions on a variety of physiological and affective processes [3–6]. Both pre-and post-synaptic 5-HT1A receptors contribute to the anxiety phenotype in mouse models [7]. Global knockout of the 5-HT1A receptor or early postnatal knockdown of the pre-synaptic 5-HT1A autoreceptor results in an anxiety phenotype [8], which is rescued by expression of the post-synaptic forebrain 5-HT1A receptor in the global 5-HT1A-/- mice [9]. On the other hand, a 30 % knockdown of presynaptic 5-HT1A receptors in the adult results in resilience to stress and depression [10]. One possible explanation is that 5-HT hyperactivity during development leads to anxiety, while a mild increase in 5-HT activity in adult leads to resilience to depression. Conversely, an increase in 5-HT1A autoreceptors would lead to increased susceptibility to depression by reducing serotonergic activity [3–6]. Thus, subtle alterations in the expression of presynaptic 5-HT1A receptors at different stages of life can significantly influence behavior phenotypes.

In humans, altered activity of the serotonin system has long been implicated in major depression and anxiety disorders [11]. These illnesses are associated with alterations in cerebrospinal fluid levels of 5-HT and its metabolites, regional changes in 5-HT receptors, and functional polymorphisms that alter expression of 5-HT genes [12, 13], including the 5-HT1A C (-1019) G rs6295 [14]. The risk allele G (-1019) fails to bind to transcriptional repressors, including NUDR/Deaf1. Deaf1 is a transcription factor that has been shown to repress its own expression, as well as that of several target genes, including the 5-HT1A receptor [15, 16]. We have shown that the G (-1019) prevents Deaf1 repression of the 5-HT1A gene, resulting in increased transcription of the 5-HT1A autoreceptor [16]. Consistent with these findings, in human depressed subjects, the G (-1019) allele has been associated with increased raphe 5-HT1A binding potential [17]. However, these associations in human subjects are not always observed, suggesting that adaptive changes in 5-HT1A autoreceptor function may compensate for the risk alleles [18, 19]. Furthermore, these illnesses are treated with antidepressant drugs that enhance 5-HT neurotransmission, but chronic treatment is required despite their rapid entry in the brain, suggesting a role for adaptive changes in 5-HT1A autoreceptor function in antidepressant response [20].

In order to model the effect of the 5-HT1A G (-1019) allele and the role of Deaf1 *in vivo*, we have studied the Deaf1 knockout mouse. In Deaf1-/- mice on a C57BL6 background, raphe 5-HT1A RNA and 5-HT1A autoreceptor-labeled cells were markedly increased, with a small reduction in 5-HT1A RNA and cells labeled for post-synaptic 5-HT1A receptors in the prefrontal cortex [21]. However, the behavior of these mice could not be addressed due to lethality from partially penetrant exencephaly [22]. To avoid this lethality, Deaf1-/- mice were bred on a mixed C57BL6-BALB/c background. Consistent with our previous study [21], the knockout of Deaf1 led to an increased level of 5-HT1A autoreceptor binding in the raphe and to an exaggerated autoreceptor-mediated hypothermia response. However, the up-regulation of 5-HT1A autoreceptor function following Deaf1 gene deletion waned with successive generations, as determined by both 5-HT1A-mediated hypothermia and cellular electrophysiology from 5-HT neurons. Knockout of Deaf1 led to a sex-dependent effect with an initial increase in hypothermia response and anxiety in males that declined additional generations, while females had normal autoreceptor function and a partial anxiety effect. These studies suggest that adaptation in 5-HT1A receptor function in Deaf1-/- mice is sex-dependent, and the anxiety phenotype persists but differs in male and female Deaf1-/- mice.

## Results

### Adaptive changes in 5-HT1A autoreceptor responses in Deaf1 knockout mice

**Fig. 1:**
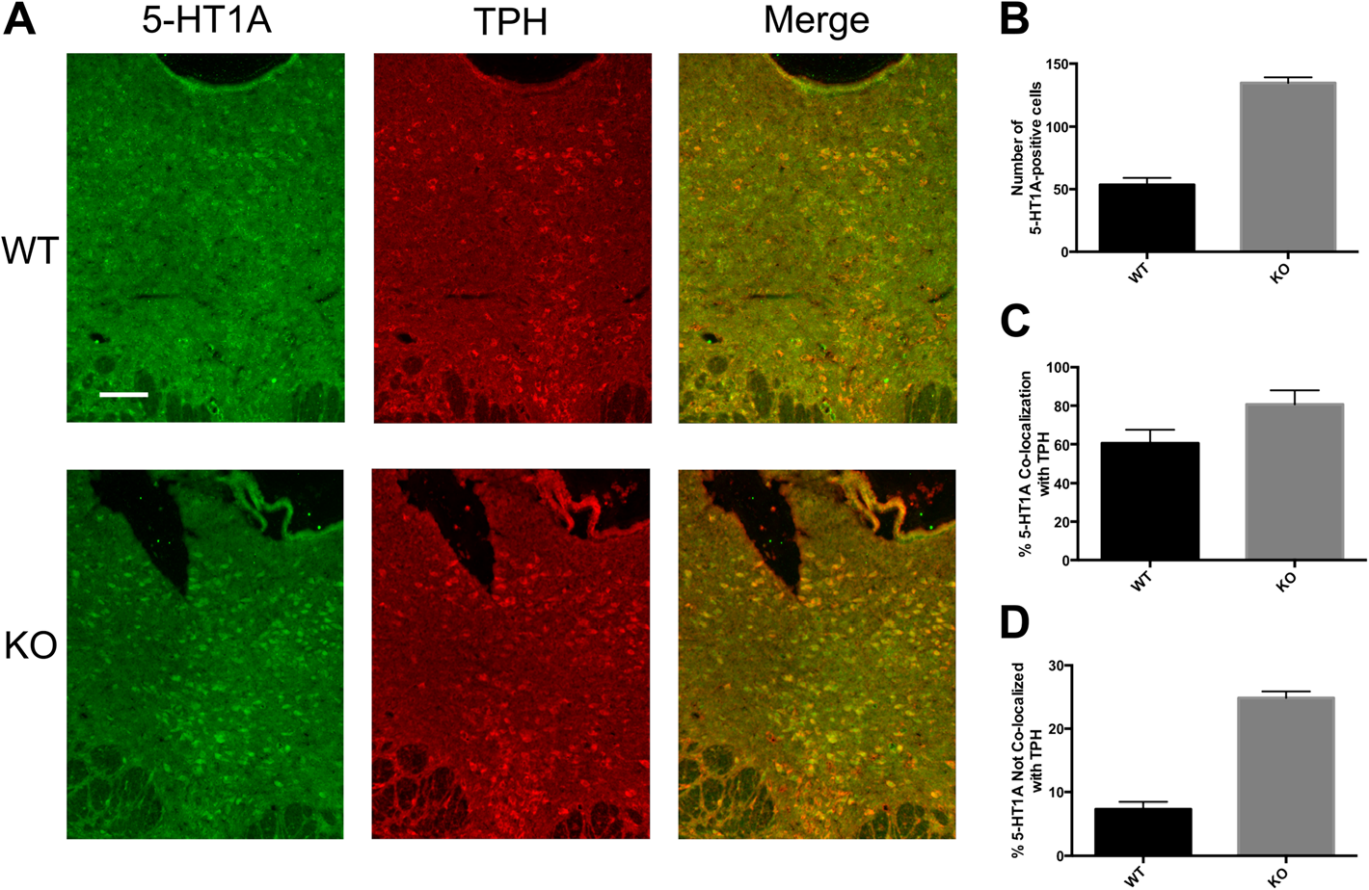
Increased 5-HT1A receptors in dorsal raphe of Deaf1-/-mice. **a **Immunofluorescent staining for TPH (sheep anti-TPH, 1:100) and 5-HT1A receptors (rabbit anti-5-HT1A, 1:50) was performed on dorsal raphe slices of male wild-type (WT) and homozygous Deaf1-/- (KO) C57BL/6-BALB/c mice. Scale bar indicates 100 μm. **b**-**d** Quantification of total **b**, TPH-positive **c** and TPH-negative **d** cells with 5-HT1A receptor staining. Mean ± SE of 2 independent counts

**Fig. 2:**
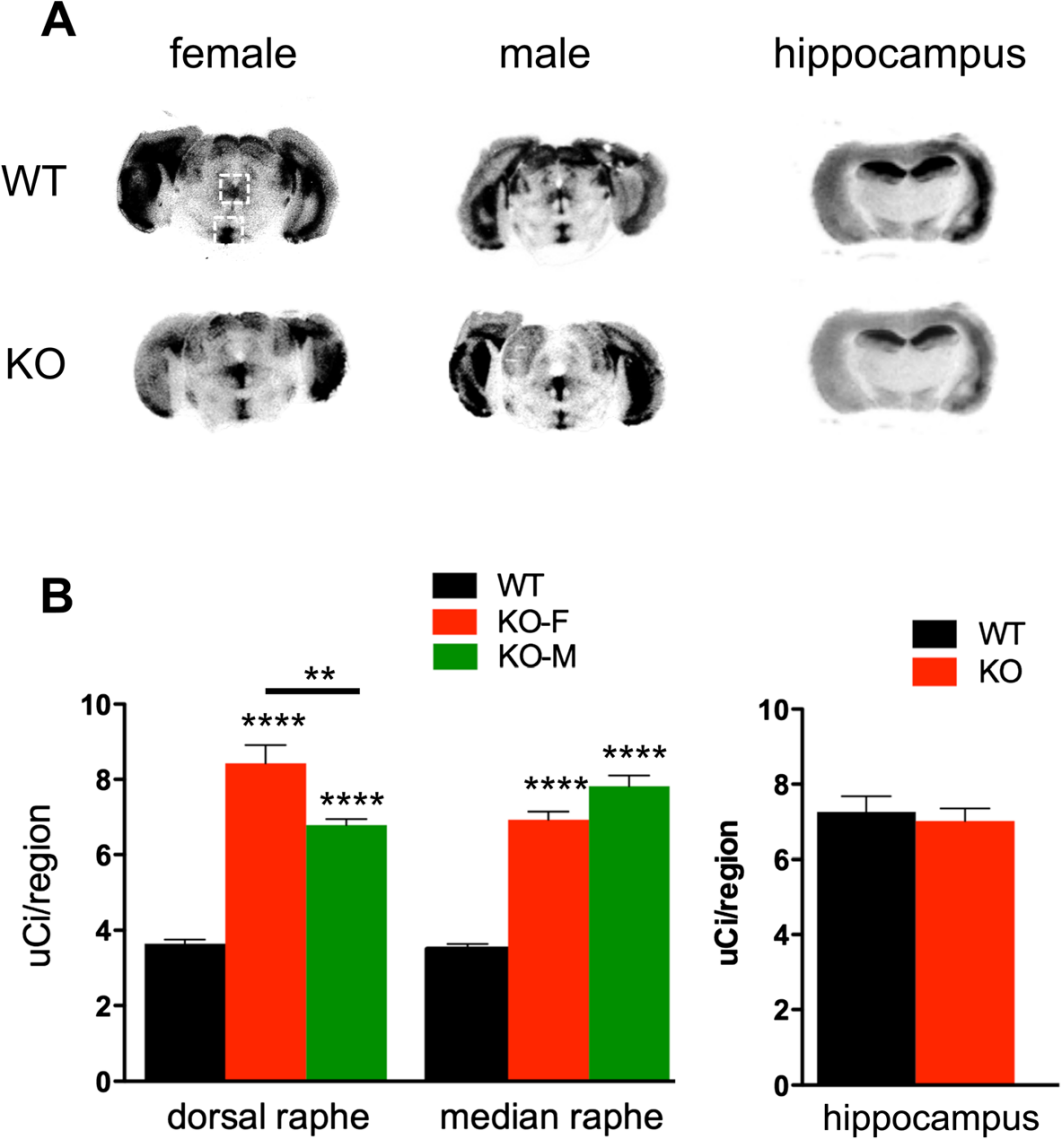
Increased 5-HT1A receptor binding in Deaf1-/- raphe. 5-HT1A receptor autoradiography of representative midbrain (male-M and female-F) and hippocampus (male) sections from Deaf1-/- (KO) and wild-type (WT) mice was done using ^125^I-MPPI. **a** Representative sections of raphe, including dorsal and median raphe (in boxes), and of hippocampus are shown. **b** Average signal/region was quantified as described in Methods for dorsal and medial raphe, and hippocampus. Since there was no sex difference in 5-HT1A binding in raphe (WT) and hippocampus (WT and KO), pooled values are shown. Data represent mean ± SEM (*n =* 4), **p < 0.01; ****p < 0.0001

We previously observed increased levels of 5-HT1A autoreceptors in Deaf1-/-mice on the C57BL6 background [21], and addressed whether this occurs in Deaf1-/- C57BL6 mice crossed to BALB/c mice and bred for several generations. Immunofluorescence staining of raphe sections from male mice was using antibodies to 5-HT1A receptor and TPH, a marker of 5-HT neurons (Fig. 1). There was a 2.5-fold increase in 5-HT1A and TPH co-labeled cells for the Deaf1-/- genotype and a 2-fold increase in the Deaf1 +/- mice compared to wild-type, similar to the 3-fold increase observed on the C57BL6 background [21], confirming an increase in 5-HT1A autoreceptors in the Deaf1-/- mice. In order to quantify the levels of 5-HT1A receptors, we performed autoradiography using the selective 5-HT1A antagonist ^125^I-MPPI [23] (Fig. 2). In both male and female Deaf1-/- mice, a significant increase in 5-HT1A receptor binding sites was observed in the dorsal (greater in females) and median raphe compared to wild-type mice, with no significant change in hippocampal 5-HT1A receptor levels. These results clarify that in both male and female mice, knockout of Deaf1 results in a specific up-regulation of 5-HT1A autoreceptors.

**Fig. 3:**
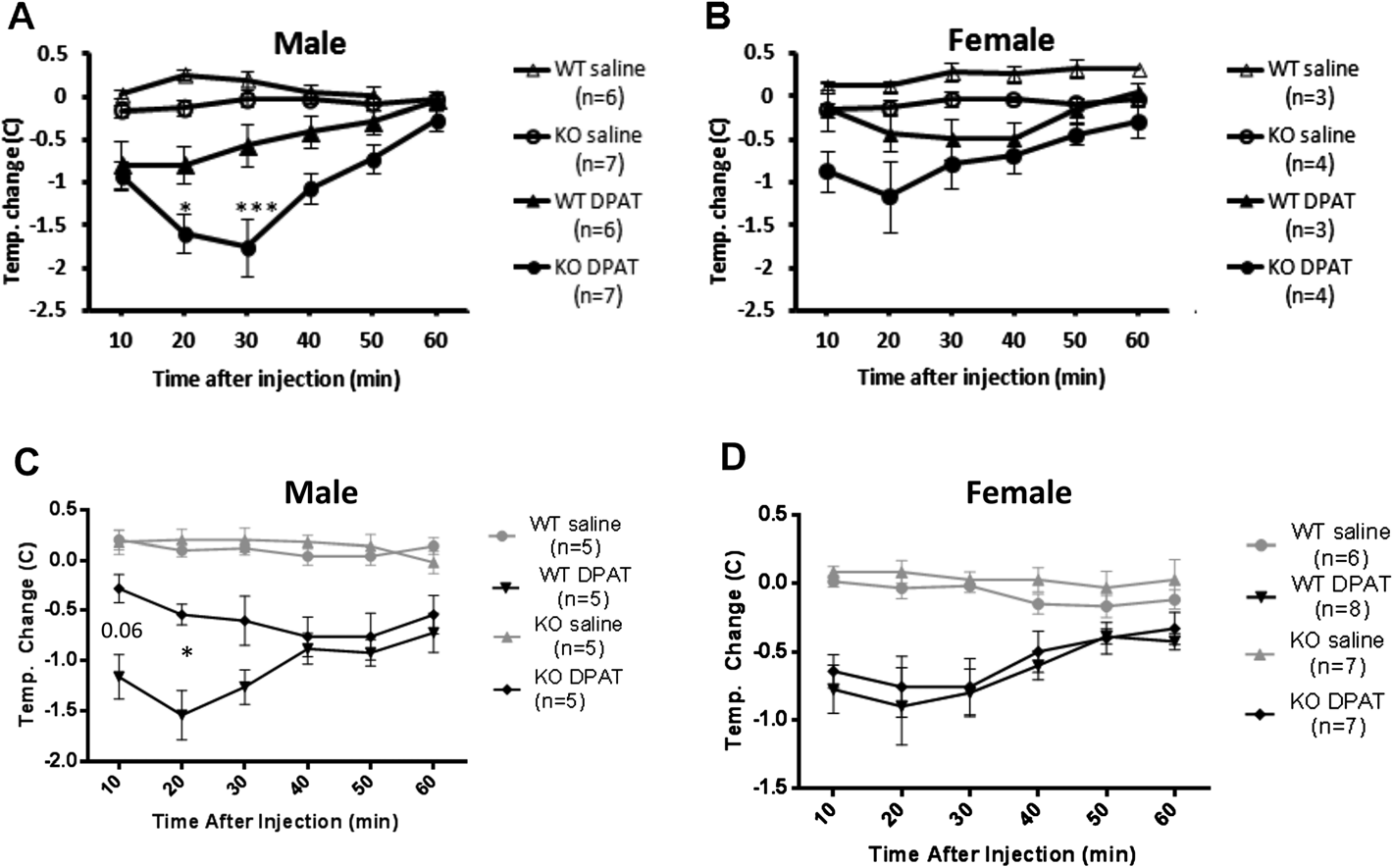
DPAT-induced hypothermia in Deaf1 knockout mice. **a**, **b** Early generation (<2) male Deaf1-/- mice show enhanced DPAT-induced hypothermia. Average body temperature change over time for male **a** or female **b** Deaf1 wild-type (WT) and Deaf1 knockout (KO) on C57BL/6-BALB/c background following 0.9 % saline or 0.5 mg/kg 8-OH-DPAT (DPAT) injection. *p < 0.05; **p < 0.01; ***p < 0.001 vs. wild-type. **c**, **d** Reduced DPAT-induced hypothermia in late generation (≥3) Deaf1-/- male mice. Data are presented as mean ± SE; *p < 0.05; ***p < 0.001 vs. wild-type

To examine whether the increased level of 5-HT1A receptors results in greater functional activity, the magnitude of hypothermia induced by acute administration of the 5-HT1A agonist DPAT was measured as an index of 5-HT1A autoreceptor responsiveness *in vivo* (Fig. 3) [8]. In first generation mixed background mice, we observed a significant 3-fold greater reduction in body temperature in the Deaf1 knockout males compared to wild-type males and a smaller enhancement in females, at 0.5 mg/kg DPAT (Fig. 3a, b) and 0.25 mg/kg DPAT (not shown). In heterozygotes, the DPAT response did not differ from wild-type mice (not shown). Unexpectedly, in later (≥3) generations on the mixed background, despite the increase in 5-HT1A autoreceptor levels (Figs. 1, 2), the DPAT-induced hypothermia was no longer enhanced in Deaf1-/- females, and was actually attenuated in male Deaf1 knockout mice compared to wild-type mice at 0.5 mg/kg (Fig. 3c, d) and 0.25 mg/kg DPAT (not shown). These data suggest that an increase in 5-HT1A autoreceptor function observed in the first generation of Deaf1-/- C57BL6-BALB/c mice is compensated for by adaptation of 5-HT1A receptor responsiveness in succeeding generations.

**Fig. 4:**
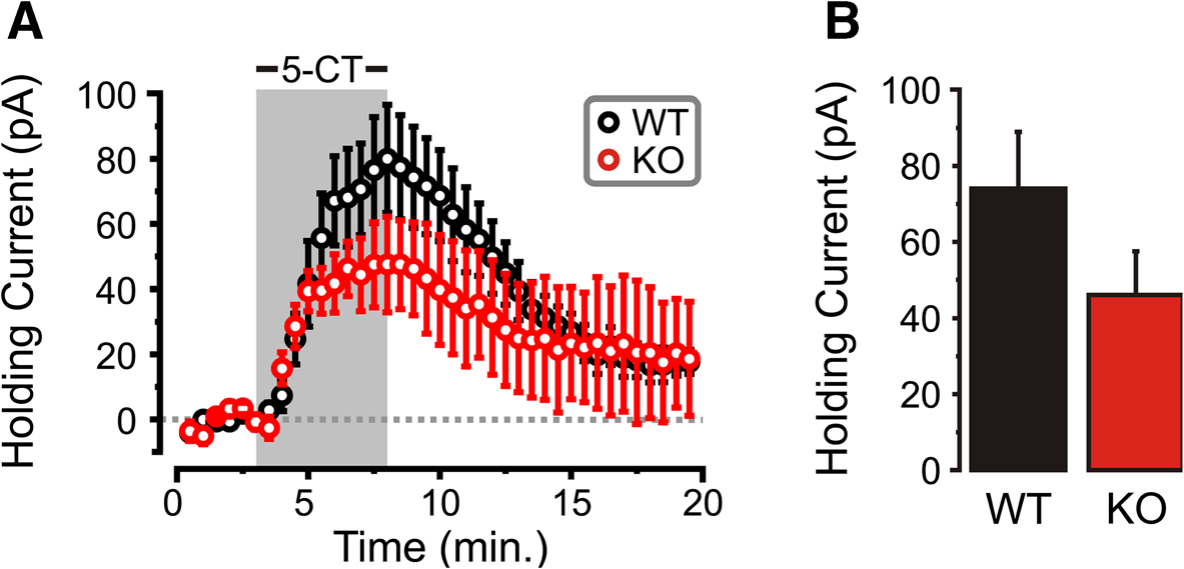
5-HT1A-induced outward currents are unchanged in Deaf1-/- mice. Whole-cell voltage-clamp recordings of DRN 5-HT neurons from wild-type and Deaf-/- mice (*n =* 7 (3 male/4 female) and 6 (2 male/4 female) cells, respectively). **a** Time Course: 5-HT1A receptor-mediated outward current in response to 5-CT (100 nM; Vm = -55 mV). **b** 5-CT response: Average peak steady-state 5-HT1A-mediated currents from recorded 5-HT neurons in wild-type and Deaf1-/-tissues. Data are presented as mean ± SE

To further investigate the mechanisms underlying a potential adaptation of the function of 5-HT1A autoreceptors with succeeding generations of Deaf1-/- mice, we performed whole-cell recordings (Vm = 55 mV) of 5-HT neurons from late (≥ 3) generation mixed background mice and monitored the effects of bath administration of the 5-HT1A agonist 5-CT (100 nM). Recordings from raphe 5-HT neurons revealed 5-HT1A receptor-mediated outward currents of similar magnitude in Deaf1 knockout and wild-type mice (Fig. 4). We further examined our data set for potential sex-specific alterations in the responsiveness of 5-HT1A receptors and found that the maximum 5-CT induced current was reduced by 60 % in Deaf1 KO compared to wild-type mice, with no difference observed in females (Additional file 1: Figure S1). These results are consistent with the hypothermia data (Fig. 3), and together indicate that the functional activity of 5-HT1A autoreceptors present in Deaf1-/-mice actually declines over generations, despite increased abundance of 5-HT1A autoreceptors.

### Anxiety phenotypes in late generation male and female Deaf1-/-mice

**Fig. 5:**
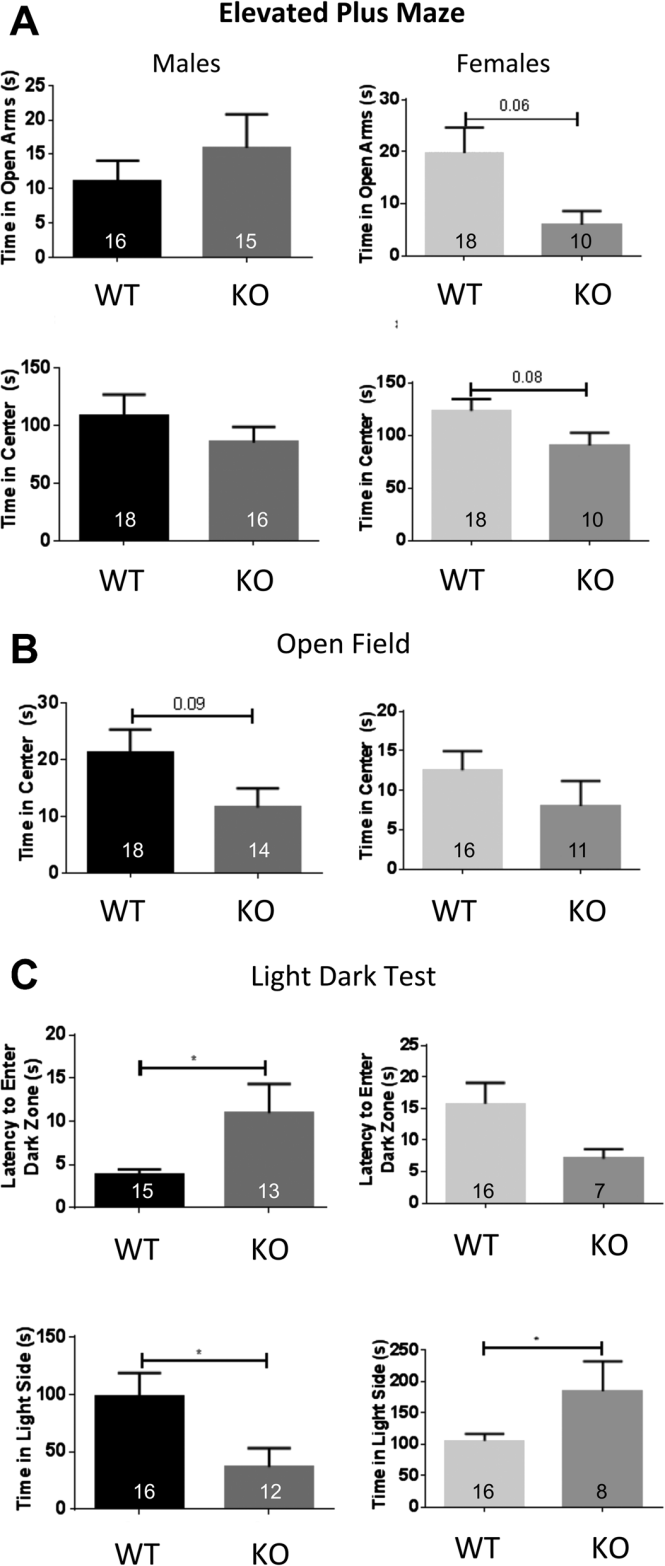
Anxiety-like behavior in Deaf1 knockout mice. Anxiety phenotype was assessed using three tests in male (left panels) and female (right panels) wild-type (WT) vs. Deaf1 knockout (KO) mice, N values indicated in panels. **a** Elevated Plus Maze test. Time spent in open arms, in the center and total distance travelled were averaged. **b** Open Field test. Time spent in the center and total distance traveled. **c** Light-Dark test. Time spent in the light side and latency to enter the dark zone for the first time was averaged. Data are presented as mean ± SE; *p < 0.05 or as indicated

In order to address the effects of Deaf1 gene deletion on behavior, we performed pilot experiments testing locomotor activity, anxiety, and depression phenotypes in late generation mice and observed differences between males and females (Additional file 1: Figure S2). Therefore, the behavior of male and female Deaf1 deficient mice was examined separately. Anxiety-like behavior was measured using three well-validated tests: the elevated plus maze (EPM), open field (OF), and light-dark tests (Fig. 5). In the EPM, time spent in open arms was not different in male Deaf1-/- mice compared to wild-type mice (Fig. 5a). However, in the open field test, there was a trend for male Deaf1-/- mice to avoid the centre (Fig. 5b). In the light-dark test, male Deaf1-/- mice showed reduced time in the light and had significantly increased latency to enter the dark chamber, indicating a freezing response when initially placed in the light, both consistent with an anxiety phenotype (Fig. 5c). Overall, two of three tests indicated that male Deaf1-/- mice tend to have higher anxiety. Female Deaf1-/- mice showed a 75 % reduction in time in open arms in the EPM compared to wild-type mice, suggesting an anxiety phenotype in this test (Fig. 5a). In contrast, no increase in anxiety was observed in female Deaf-/- mice in the OF (Fig. 5b) or the light dark-test (Fig. 5c). Specifically, in the light-dark test the female knockouts spent more time in the light, suggesting reduced anxiety (Fig. 5c). These differences seen in anxiety tests did not appear to be caused by any locomotor impairment since there were no differences between genotypes or sex in novel cage activity (Additional file 1: Figure S3). Taken together, the anxiety-like behavior in Deaf1-/- mice was sex-dependent: the most prominent anxiety phenotype occurred in light-dark test for males, while in the EPM for female Deaf1-/- mice.

**Fig. 6:**
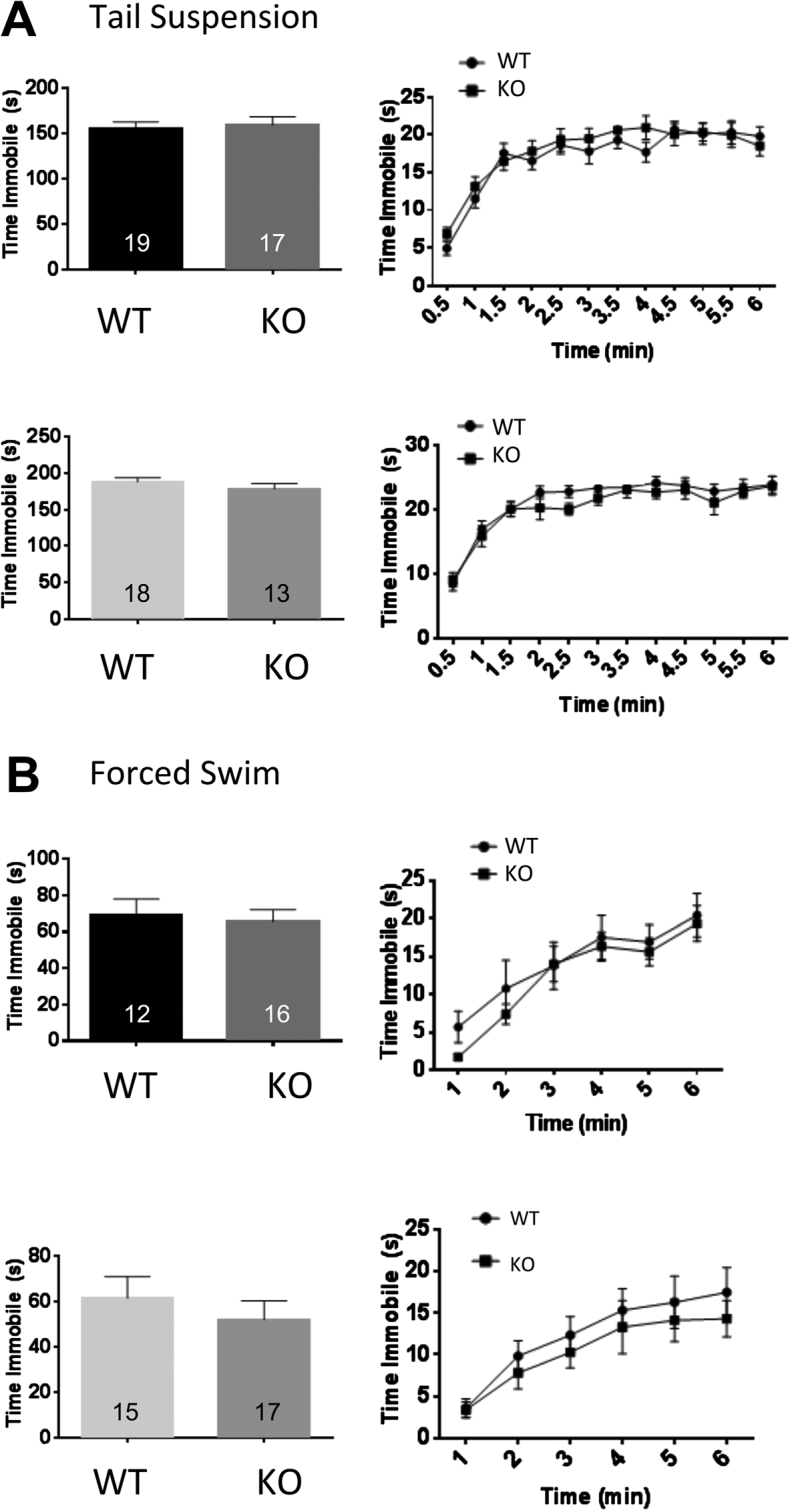
Depression-like behavior in Deaf1 knockout mice. Behavioral despair phenotype was assessed using tail suspension and forced swim tests in male (upper panels) and female (lower panels) wild-type (WT) and Deaf1 knockout (KO) mice, N values as indicated. **a** Tail suspension test. Time spent immobile was averaged across the final four minutes of the test (left panels) and the time spent immobile shown in 30-second intervals across the full 6-min duration (right panels). **b** Forced Swim test. Time spent immobile averaged across the final four minutes of the FS test (left panels). Time spent immobile shown in 30-second intervals across the full 6-min FS test (right panels). Data are presented as mean ± SE

When tested in two different assays for depression-like despair behavior, the tail suspension test (Fig. 6a) and forced swim test (Fig. 6b), Deaf1-/- male and female mice were indistinguishable from wild-type mice. These results indicate a lack of depression-like phenotype in Deaf1 knockout mice. Similarly, in preliminary studies no significant difference between wild-type or Deaf1 knockout mice was seen in the sucrose preference test (data not shown). These data suggest that the Deaf1-/- mice have sex-dependent changes in anxiety, but no detectable depression-like behavior.

## Discussion

### 5-HT1A autoreceptor adaptation in Deaf1-/-mice

Deaf1 has been implicated in the regulation of 5-HT1A receptor expression *in vitro*, and indirectly in major depression in humans [16, 24]. Deaf1 binds to sites in the human and mouse 5-HT1A promoters to repress 5-HT1A transcription in raphe and non-neuronal cells. However, in the human HTR1A promoter, Deaf1 fails to bind to its site at the rs6295 G-allele, leading to increased 5-HT1A autoreceptor expression to reduce serotonergic activity and predispose to depression. The G-allele has been associated with major depression and suicide [16, 18, 25], and with increased 5-HT1A autoreceptor levels in depressed patients [26, 27]. Based on its repressor activity at the mouse 5-HT1A promoter, we found that knockout of Deaf1 in mice increases 5-HT1A autoreceptor expression [21], which we confirm and extend here in a mixed C57BL6-BALB/c background by quantifying 5-HT1A autoreceptor binding levels (Figs. 1, 2). We therefore addressed whether the increase in 5-HT1A autoreceptors in Deaf1 knockout mice leads to functional changes *in vivo*.

We first measured DPAT-induced hypothermia, which in mice provides a read-out of the activity of raphe 5-HT1A autoreceptors *in vivo* [8, 28]. Male, but not female Deaf1-/- mice displayed enhanced DPAT-induced hypothermia in early generations, consistent with an increase in 5-HT1A autoreceptor function. However, this effect was lost in subsequent generations, with males showing a significantly blunted response to the agonist. This reversal of the DPAT response suggests that adaptations in 5-HT1A autoreceptor function occur over generations in the mixed background of male Deaf-/- mice, but the mechanisms involved remain unclear. Whole-cell recordings from 5-HT neurons in raphe slices showed that the magnitude of the outward current induced by the 5-HT1A agonist 5-CT was similar or even slightly reduced in Deaf-/- compared to wild-type slices. This result confirms that following the initial increase in 5-HT1A autoreceptor function detected in the hypothermia test, the receptor adapts its signaling to normalize effects on 5-HT neuron excitability. Multiple mechanisms could mediate the normalization of 5-HT1A autoreceptor signaling with passing generations. Compensatory reduction in coupling of 5-HT1A autoreceptors to G-proteins and GIRK channels could reduce 5-HT1A responses, as observed following chronic stress or corticosterone treatment [29–31]. Thus, homeostatic mechanisms appear to compensate for the up-regulation of 5-HT1A autoreceptors to normalize 5-HT1A receptor function, which could account for the relatively mild behavioral phenotype observed in the Deaf1-/- mice.

The mixed C57BL6-BALB/c background could enhance the normalization of 5-HT1A autoreceptor responses in Deaf1 null mice, since BALB/c mice have a mutation in the TPH2 gene that reduces 5-HT synthesis [32]. In C57BL6 mice carrying this TPH2 mutation, DPAT-induced hypothermia was significantly reduced, despite normal 5-HT1A autoreceptor binding and G-protein coupling, implying that reducing 5-HT levels impairs the effect of DPAT or 5-HT1A responsiveness *in vivo* [33, 34]. In addition to reduced 5-HT due to the Balb/c background, the Deaf11-/- C57BL6 mice also have a 50 % reduction in 5-HT [21] which together may contribute to blunt 5-HT1A autoreceptor responsiveness on the mixed background over time. On the other hand, in 5-HTT-/- mice persistently enhanced 5-HT neurotransmission results in greater 5-HT1A receptor desensitization in females vs. males, which involves receptor down-regulation and the uncoupling of Gi proteins from GIRK channels [35–37]. Thus, in female 5-HTT-/- mice the 5-HT1A autoreceptor appears to desensitize with increased 5-HT signaling, while in male Deaf1-/- and TPH2 mutant mice, long-term reduction in 5-HT results in reduced 5-HT1A autoreceptor function.

The mixed C57BL6-BALB/c background may also contribute to the transgenerational change in 5-HT1A autoreceptor function in Deaf1 knockout mice. Non-genetic transgenerational transmission of the anxiety phenotype of the 5-HT1A knockout has been shown in mice with an outbred Swiss-Webster background, but not for inbred C57BL6 mice [38]. Embryonic implantation of wild-type 5-HT1A embryos in 5-HT1A-/- Swiss-Webster mothers transmitted the anxiety phenotype of the knockout mother to the adult offspring, and also influenced their hippocampal development. Like the 5-HT1A knockout and loss of Deaf1 in mice, the C (-1019) G HTR1A polymorphism in humans dys-regulates the HTR1A gene globally [27]. Our results in the mixed C57BL6-Balb/c background suggest that altered signaling of 5-HT1A receptors could be one mechanism of adaptation of the anxiety phenotype to the loss of Deaf1 across generations.

A transgenerational adaptation in 5-HT1A receptor function in humans could explain in part inconsistencies in the association of the 5-HT1A C (-1019) G polymorphism with major depression and anxiety in different studies [18]. The association of the 5-HT1A G (-1019) allele with major depression appears most robust in genetically homogeneous populations, such as in depressed families from Utah [39]. In females from the Utah cohort, carrying 5-HT1A risk allele strengthened association of depression with the LHPP gene, a gene also identified by genome-wide association in a depressed Chinese female population [40]. LHPP can dephosphorylate histidine residues, which could inactivate G-protein and potassium channel function to uncouple 5-HT1A receptors [41]. Greater adaptive changes in 5-HT1A function in male G (-1019) carriers could in part account for the lack of this association in male from the Utah cohort. On the other hand, increased raphe 5-HT1A binding potential was strongly associated with depression in men but not women [42], and could reflect inadequate normalization of 5-HT1A autoreceptor expression in depressed males. Thus, 5-HT1A autoreceptor regulation appears to be different in males and females and could result in differences in susceptibility to depression.

### Different phenotypes in male and female Deaf1-/- mice

Consistent with altered 5-HT1A autoreceptor function in male Deaf1-/- mice, anxiety-like behaviors differed in male and female Deaf1-/- mice. Male Deaf1-/- mice demonstrated greater anxiety-like behavior in two tests (light-dark and open field), but not in the elevated plus maze test. This is consistent with previous findings in brain-specific male Deaf1 knockout C57BL6 mice, which showed a mild anxiety phenotype in this test with no change in open arm time [22]. The female Deaf1-/- mice displayed a mixed phenotype, mildly anxious in the elevated plus maze, but with apparently reduced anxiety in the light-dark test. The elevated plus maze test is more dependent on exploratory behavior and less sensitive to neophobia than the open field or light-dark tests [43]. Hence, the weak anxiety phenotype in females may reflect reduced exploration, while the greater latency of the Deaf1-/- males to escape to the dark side in the light-dark test could be interpreted as a light-dependent panic response. In contrast, Deaf1-/- females displayed apparently reduced anxiety in the light-dark test, reduced latency to enter the dark chamber suggesting that they are less sensitive to light than the males in this test, which has the brightest light intensity of the tests. The anxiety phenotype in Deaf1-/- males is consistent with the role in males of reduced 5-HT1A autoreceptor function during the early post-natal period in the anxiety phenotype [8], that remains to be addressed in females.

The Deaf1-/- mice did not display depression-like behavior in the forced swim or tail suspension tests. Importantly, the anxiety phenotype of our mixed background Deaf1 null mice is consistent with the brain-specific knockout of Deaf1, which in male mice induced an anxiety phenotype in one test, and no depression-like effects [22]. Adaptive changes in 5-HT1A autoreceptor function in Deaf1-/- mice may blunt changes in depression-like behavior in these mice. In humans, analogous adaptive changes may explain why the association of the C (-1019) G HTR1A polymorphism with depression in humans is not always shown. Since this polymorphism affects multiple factors in addition to Deaf1, including Hes1/5 [44], additional compensatory changes may occur in humans that are not seen in Deaf1-/- mice.

In summary, our data indicate that Deaf1 regulates 5-HT1A autoreceptors *in vivo* and show that upon loss of Deaf1 regulation, compensatory changes occur over generations leading to adaptation of receptor responsiveness that may underlie the mild anxiety phenotype. Interestingly, the sex differences we observe in 5-HT1A autoreceptor function suggest that different adaptive mechanisms are recruited in males and females to regulate 5-HT activity and behavioral phenotype. Further studies comparing male and female gene regulatory mechanisms are needed to understand the generation of sex dependent differences in behavior.

## Methods

### Animals

Deaf1-/- mice on C57BL6 background were mated to BALB/c (Charles River Laboratories, Montreal, Canada) and then propagated by crosses between heterozygous Deaf1 mice. Mice were pair-housed in standard Plexiglas cages on a 12/12 h light/dark cycle with ad libitum access to food and water.

### Deaf1 genotype

DNA was extracted (REDExtract-N-Amp Tissue PCR kit, Sigma) from tissue samples and genotyped using Phusion DNA polymerase (Thermo-Fisher, Ottawa ON) and the primers: 5′-GGG CTT CCG GGT CAT TCT GT-3′, 5′-ACT AAG AGG GTC ACA CAA AAG AAC AAA-3′, and 5′-TGC ACC CAC CAC CAA GAT AAG AA-3′. The PCR conditions were: 98 °C, 30 s; 34 cycles: 98 °C, 10 s, 62 °C, 30 s, 72 °C, 20 s, 84 °C, 10s; then 72 °C, 10 min; 10 °C. This protocol results in 267-bp (wild-type) or 451-bp (knockout) products. Each mouse was genotyped both at weaning and at sacrifice.

### Immunofluorescence

Mice were anaesthetized (Euthanyl; 0.01 mL/g), perfused by cardiac infusion of PBS followed by 4 % paraformaldehyde. Whole brains were extracted and post-fixed overnight in 4 % paraformaldehyde. Brains were then kept in 20 % sucrose solution, changed daily, for five days and frozen. Coronal brain slices (12-μm) were taken from the brainstem including the dorsal raphe nuclei (DRN, Bregma -4.2 to -4.96). Slices were thaw-mounted on Superfrost slides (Thermo-Fisher) and kept at -80 °C. Staining was done for 5-HT1A receptors (purified anti-5-HT1A 1:50 [21], donkey anti-rabbit 1:1000) colocalized with TPH (sheep anti-TPH 1:100, donkey anti-sheep 1:200). Images were acquired using an Axiovert S100 Zeiss microscope. Positively-labelled cells were manually counted using ImageJ software with counter blinded to genotype.

### 5-HT1A receptor autoradiography

Deaf1 KO and WT littermate mice (11 wks old, *n =* 4/genotype) were sacrificed by cervical dislocation and decapitation. Extracted brains were frozen immediately on dry ice (-75 °C) and maintained at -80 °C until sectioning. Brains were cryosectioned at a thickness of 25 μm and mounted sections were maintained at -80 °C until processing. Mounted sections were processed for ^125^I-MPPI (Perkin Elmer, Boston, MA) autoradiography as described [23]. Sections were exposed to Kodak BioMax MR film (VWR) for 24 h. Films were digitized at 1200-dpi resolution using an Epson Perfection V500 Photo Scanner, and signal density was measured using the mean luminosity function in ImageJ (1.49). Levels of 5-HT1A binding (μCi) were quantified by analyzing a standardized template outlining the region-of-interest, and adjacent background lacking specific binding subtracted. For raphe, data from sections at Bregma -4.36, -4.48, -4.60, -4.72 cm were averaged; for hippocampus, Bregma -1.82 cm was used. Signals were within the linear range of the film and quantified based on standard curve using ARC146-F ^14^C standard (American Radiochemicals Inc, St. Louis, MO).

### DPAT-Induced Hypothermia

The procedure was performed between 11 am and 4 pm. Mice were transported to the testing room and weighed. Internal temperature was taken once every 10 min using a rectal thermometer for 40 min (4 baseline measurements) followed by intraperitoneal injection of the indicated dose of DPAT (8-hydroxy-N, N-dipropyl-2-aminotetralin; Sigma) or an equivalent volume of 0.9 % saline (vehicle). Saline injection was given to each mouse on Day 1, and DPAT injection on Day 3. For analysis purposes, the first baseline temperature was discarded. The remaining three baseline values were averaged and the difference between the average baseline and recorded temperature was graphed across time.

### Acute brain slice preparation

Brainstem slices (300-μm) containing the DRN were prepared from 8–10 week old mice as previously described [45]. In brief, mice were anesthetized and sacrificed by decapitation. Once the brain was removed, coronal slices were made from a block of brain tissue while emerged in ice-cold choline chloride-based cutting solution of the following composition: (mM): 119 choline-Cl, 2.5 KCl, 1 CaCl_2_, 4.3 MgSO_4_-7H_2_O, 1 NaH_2_PO_4_, 1.30 sodium L-ascorbate, 26.20 NaHCO_3_, and 11 glucose, and equilibrated with 95 % O_2_, 5 % CO_2_. Slices were then recovered in a chamber with standard Ringer’s solution (mM): 119 NaCl, 2.5 CaCl_2_, 1.3 MgSO_4_-7H_2_O, 1 NaH_2_PO_4_, 26.2 NaHCO_3_, and 11 glucose, and bubbled with 95/5 % O_2_/CO_2_.

### Whole-cell electrophysiology

DRN neurons were visualized using an upright microscope (Examiner D1; Zeiss, Oberkochen, Germany) equipped with Dodt-gradient-contrast (40×/0.75NA objective). 5-HT neurons were identified by morphological and biophysical characteristics as previously established (Geddes, et al., 2015). Whole-cell recordings carried out at room temperature in standard Ringer’s solution using borosilicate glass patch electrodes (3–6 MΩ; World Precision Instruments). 5-HT_1A_ receptor-mediated currents were elicited by bath applying the 5-HT_1A_ receptor agonist 5-carboxamidotryptamine (5-CT; 100 nM; Tocris) when recording from 5-HT neurons in the DRN. Holding current was monitored at 0.1 Hz while the cells were voltage-clamped at -55 mV. These recordings were carried out using an internal solution of the following composition (mM): 115 potassium gluconate, 20 KCl, 10 sodium phosphocreatine, 10 HEPES, 4 Mg^2+^-ATP, and 0.5 GTP, pH 7.25 (adjusted with KOH; osmolarity, 280-290 mOsmol/L). Access resistance was continuously monitored by applying a 125 ms, 2 mV hyperpolarizing pulse every 10 s, and recordings were discarded if the access resistance changed by >30 %.

### Behavioral testing

All tests were performed in the University of Ottawa Behavioral Core Facility using 8–9 week old Deaf1-/- and WT littermate mice between 9 AM and 5 PM. The tests were performed in the following order: elevated plus maze, open field, light-dark paradigm, tail suspension, and forced swim test, with a minimum of 2 days between tests. The beam break test was performed after elevated plus maze and before the tail suspension test. Apparati were cleaned after each trial.

### Elevated plus maze

Each mouse was placed in the center of an elevated, two-arm plus maze (Noldus, The Netherlands) measuring 6 cm wide and 75 cm long and 74 cm above ground for 10 min with illumination (100–110 Lux). The closed arm was enclosed by walls 20 cm tall while the open arms had no walls. Open and closed arm time was monitored by overhead camera, fed to a computer, and analyzed using Noldus Ethovision (version 10) software.

### Open field test

The mouse was placed in the corner of an opaque, illuminated (250–300 Lux) white box measuring 45 × 45 × 45-cm for 10 min and monitored using an overhead camera fed to a computer in a separate room and analyzed using Noldus Ethovision 10 software. The time spent outside of a 24 × 24-cm square in the center of the box was used to assess anxiety.

### Light-dark test

The mouse was placed in the corner of the light compartment (390 lux) of the light-dark box (Med Associates Inc., St. Albans, VT) that had an opening that allowed the mouse to freely move between light and dark compartments and monitored for 10 min. The dark compartment was of equal size and was covered by a black, opaque insert. Movement within the compartments was detected using infrared transmitters and receivers positioned around the chamber periphery.

### Tail suspension test

The mouse was taped by its tail to an aluminum bar attached to a transducer for 6 min and mobility measured as movement above a set threshold, as recommended (Med Associates Inc., St. Albans, VT).

### Forced swim test

The mouse was placed in a clear, plexiglass cylinder (diameter, 22 cm; height, 37 cm) filled to 5–10 cm from the top with 23–25 °C water for 6 min under red light. Movement was monitored using a camera placed in front of the cylinder and mobility/immobility time analyzed using Med Associates' software.

### Locomotor activity

Each mouse was placed in a novel cage within a metal frame equipped with infrared detectors (Micromax) and beam breaks were recorded over 2 h (Micromax, Omnitech Electronics Inc., Columbus, OH).

### Statistical analyses

All analyses were done using the Statistical Package for the Social Sciences (GraphPad Prism version 6.00 for Windows, GraphPad Software, La Jolla, CA, www.graphpad.com). Data are expressed as mean ± standard error of the mean (SEM). For immunofluorescence quantification only sections falling between Bregma -4.36 to -4.96 were included in analysis. For behavior analyses, data points that lay outside ±2 standard deviations (SD) from the mean were excluded. Statistical analyses were performed using unpaired two-tailed t-test to compare data from wild-type to knockout. When comparing data across time or across gender, a 2-way repeated measures analysis of variance was used. Post hoc comparisons were made with Bonferroni multiple comparisons test.

## Abbreviations

^125^I-MPPI, 4-(2′-Methoxyphenyl)-1-[2′-(n-2′′-pyridinyl)-p-[^125^I] iodobenzamido] ethylpiperazine); 5-CT, 5-carboxamidotryptamine; 5-HT, serotonin (5-hydroxytryptamine); DPAT, 8-hydroxy-N, N-dipropyl-2-aminotetralin; EPM, elevated plus maze test; KO, knockout; LD, light-dark test; OF, open field test; WT, wild-type

## Additional file

Additional file 1: Figure S1. Sex Comparisons: Electrophysiological Recordings. Comparisons between the sex and Deaf1 genotype in whole-cell voltage-clamp recordings of DRN 5-HT neurons from wild-type (WT) and Deaf-/- (KO). Time Course (left) and average peak steady-state outward current response (right) to 5-HT1A agonist 5-CT CT (100 nM; Vm = -55 mV) are shown for WT and KO mice. a Females (n=4/genotype). b Male (n=3 or 2 for WT, KO). Data shown as mean ± SE. **Figure S2**. Sex Comparisons: Anxiety Assays. Comparisons between the sex and Deaf1 genotype (wild-type +/+ vs. knockout -/-) were made for each anxiety test to determine whether there were sex differences. Elevated Plus Maze. Time spent in the opens arms or in the center, and total distance traveled were compared. Open Field Test. Time spent in the center and total distance traveled were compared. Light-Dark Test. Time spent in the light side and latency to first enter the dark zone and total distance traveled were compared. Data shown as mean ± SE; **p*<0.05; ***p*<0.01; ****p*<0.001. **Figure S3**. Locomotor Activity. Comparisons between the sex and Deaf1 genotype were made using the Locomoter Activity Test. In male and female mice the number of movements (beam breaks) is shown as a measure of locomotor activity for the Deaf1 wild-type (+/+) and knockout (-/-). Data shown as mean ± SE. (PDF 318 kb)
