# Supplementary material for: Sex-dependent adaptive changes in serotonin-1A autoreceptor function and anxiety in Deaf1-deficient mice
Source: Mol Brain. 2016 Aug 3;9:77. doi: 10.1186/s13041-016-0254-y (PMC4973060; doi:10.1186/s13041-016-0254-y)
Supplement: Additional file 1: Figure S1. — Sex Comparisons: Electrophysiological Recordings. Comparisons between the sex and Deaf1 genotype in whole-cell voltage-clamp recordings of DRN 5-HT neurons from wild-type (WT) and Deaf-/- (KO). Time Course (left) and average peak steady-state outward current response (right) to 5-HT1A agonist 5-CT CT (100 nM; Vm = -55 mV) are shown for WT and KO mice. a Females (n=4/genotype). b Male (n=3 or 2 for WT, KO). Data shown as mean ± SE. Figure S2. Sex Comparisons: Anxiety Assays. Comparisons between the sex and Deaf1 genotype (wild-type +/+ vs. knockout -/-) were made for each anxiety test to determine whether there were sex differences. Elevated Plus Maze. Time spent in the opens arms or in the center, and total distance traveled were compared. Open Field Test. Time spent in the center and total distance traveled were compared. Light-Dark Test. Time spent in the light side and latency to first enter the dark zone and total distance traveled were compared. Data shown as mean ± SE; *p<0.05; **p<0.01; ***p<0.001. Figure S3. Locomotor Activity. Comparisons between the sex and Deaf1 genotype were made using the Locomoter Activity Test. In male and female mice the number of movements (beam breaks) is shown as a measure of locomotor activity for the Deaf1 wild-type (+/+) and knockout (-/-). Data shown as mean ± SE. (PDF 318 kb) [file 13041_2016_254_MOESM1_ESM.pdf]

Figure S1

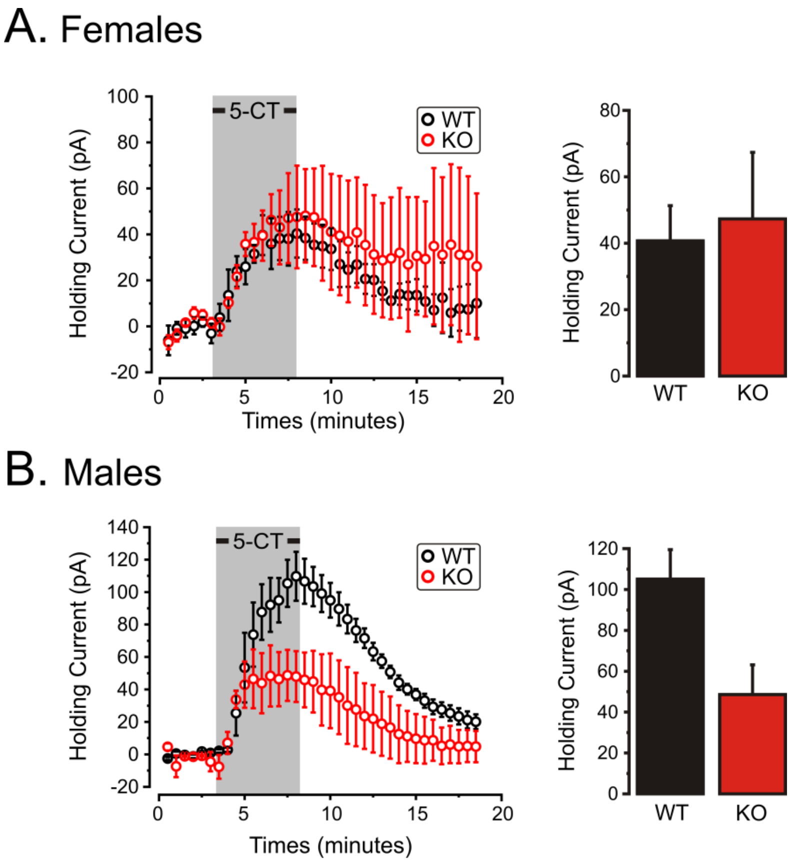

Figure S1. Sex Comparisons: Electrophysiological Recordings. Comparisons between the sex and Deaf1 genotype in whole-cell voltage-clamp recordings of DRN 5-HT neurons from wild-type (WT) and Deaf -/- (KO). Time Course (left) and average peak steady-state outward current response (right) to 5-HT1A agonist 5-CT CT (100 nM; Vm = -55 mV) are shown for WT and KO mice. **a** Females (n=4/ genotype). **b** Male (n=3 or 2 for WT, KO). Data shown as mean ± SE.

Figure S2

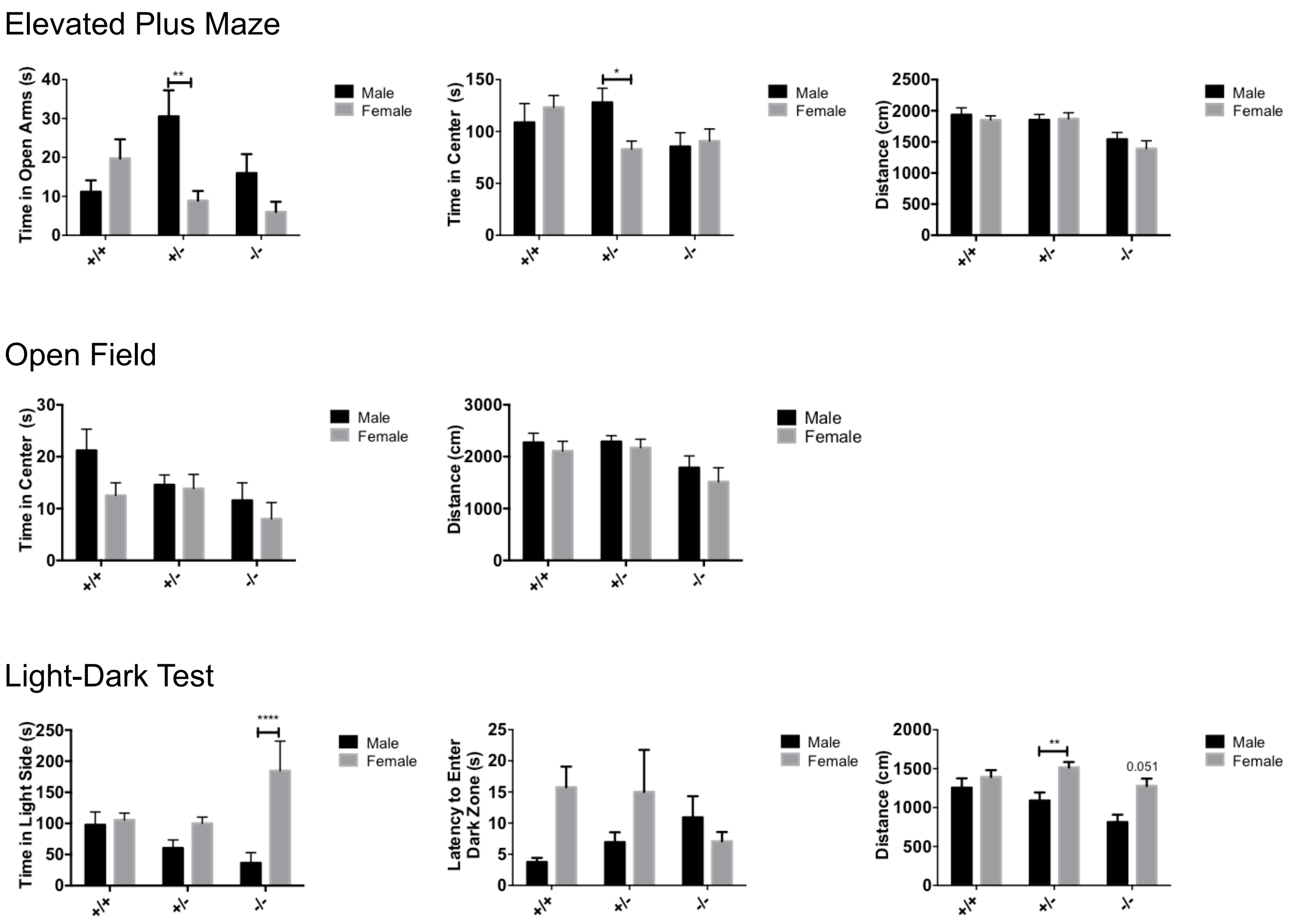

Figure S2. Sex Comparisons: Anxiety Assays. Comparisons between the sex and Deaf1 genotype (wild-type +/+ vs. knockout -/-) were made for each anxiety test to determine whether there were sex differences. Elevated Plus Maze. Time spent in the opens arms or in the center, and total distance traveled were compared. Open Field Test. Time spent in the center and total distance traveled were compared. Light-Dark Test. Time spent in the light side and latency to first enter the dark zone and total distance traveled were compared. Data shown as mean ± SE; *$p<0.05$; **$p<0.01$; ***$p<0.001$

Figure S3

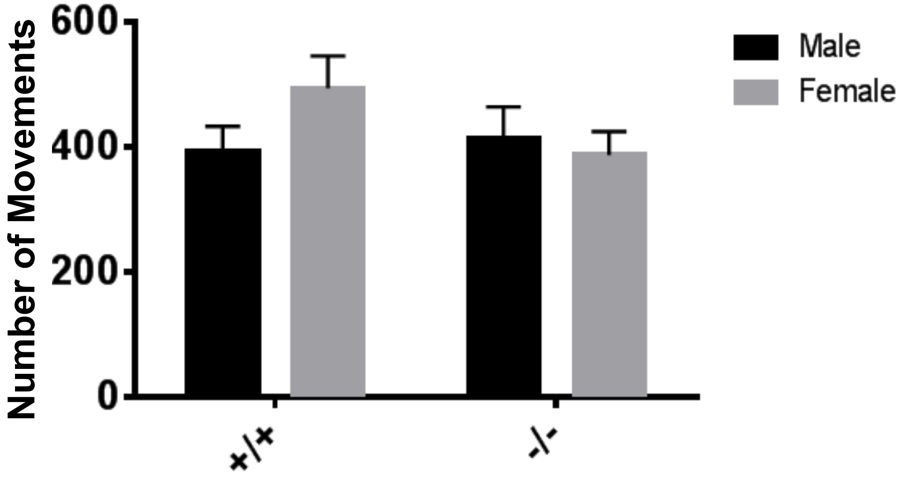

Figure S3. Locomotor Activity. Comparisons between the sex and Deaf1 genotype were made using the Locomoter Activity Test. In male and female mice the number of movements (beam breaks) is shown as a measure of locomotor activity for the Deaf1 wild-type (+/+) and knockout (-/-). Data shown as mean ± SE.
